# Data on fluoride concentration and health risk assessment of drinking water in Khorasan Razavi province, Iran

**DOI:** 10.1016/j.dib.2018.04.045

**Published:** 2018-04-21

**Authors:** Mansour Ghaderpoori, Ali Asghar Najafpoor, Afshin Ghaderpoury, Mahmoud Shams

**Affiliations:** a Department of Environmental Health Engineering, School of Health and Nutrition, Lorestan University of Medical Sciences, Khorramabad, Iran; b Nutritional Health Research Center, Lorestan University of Medical Sciences, Khorramabad, Iran; c Department of Environmental Health Engineering, School of Health, Mashhad University of Medical Sciences, Mashhad, Iran; d Social Determinants of Health research center, Mashhad University of Medical Sciences, Mashhad, Iran; e Student Research Committee, Shahid Beheshti University of Medical Sciences, Tehran, Iran

**Keywords:** Fluoride, Health risk assessment, Drinking water, Khorasan Razavi

## Abstract

While fluoride (F) is an essential anion to keep the human body healthy, high F intake could lead to serious health problems. The monitoring of F in drinking water as the main route of F intake, is a key factor in preventing its negative health consequences. Here, we present the F levels in drinking water distribution networks of Khorasan Razavi province in Iran which collected during 2016–2017. The non-cancer human risk attributed to F in municipal and rural regions, also, estimated by calculating the chronic daily intake (CDI) and hazard quotient (HQ) for adults and children. Samples taken from drinking water distribution network in 112 different locations across the Khorasan Razavi and the F concentration determined using standard SPADNS Method. Having a minimum of 0.09 and 0.16 and a maximum of 1.7 and 1.1 mg L^−1^, the mean F level in municipal and rural samples were 0.74 and 0.59 mg L^−1^, respectively. The mean CDI values for F in municipal samples were 1.3×10^−2^, 3.34×10^−4^, and 8.56×10^−6^ mg kg^−1^day^−1^, for men, women, and children, respectively. The CDI for rural samples were 1.51×10^−2^, 3.88×10^−4^, and 9.96×10^−6^ mg kg^−1^day^−^^1^, for men, women, and children, respectively. The mean HQ of F for men, women, and children in municipal and rural samples were 2.17×10^−1^, 5.56×10^−3^, and 1.43×10^−4^, and 2.44×10^−1^, 6.26×10^−3^ and 1.61×10^−4^, respectively. Locations with a HQ>1, needs appropriate strategies for reducing the F level in drinking water to prevent the potential health risks.

**Specifications table**

**Table:** 

Subject area	*Water quality assessment*
More specific subject area	*Drinking water monitoring and quality*
Type of data	*Table, figure*
How data was acquired	*The fluoride level in samples determined according to the standard methods for the examination of water and wastewater (SPADNS Method) using a DR-5000 spectrophotometer (UV–vis).*
Data format	*Raw, analyzed*
Experimental factors	*According to the study area, 112 sampling points were identified. After sampling, all samples were stored and carried to the laboratory analyzed for the concentration of fluoride.*
Experimental features	*Measuring the concentration of fluoride ion in the samples of drinking water*
Data source location	*Mashhad city, Khorasan Razavi province, east of Iran*
Data accessibility	*Data are included in this article and supplemented excel file*

**Value of the data**

• The unusual properties of fluoride (F) in having detrimental health effects at excess or deficient intake levels, give these data value to be available.
• Drinking water is the main route of fluoride intake. The role of F in keeping bones and teeth healthy is fully understood. Harmful non-carcinogenic problems related to excess F intake are skeletal fluorosis, dental fluorosis, and dental caries.
• The estimation of non-cancer human health risk assessment attributed to F for the population who consume the water, is a systematic approach for developing management strategies for supply safe drinking water.

## 1 Data

**Fig. 1:**
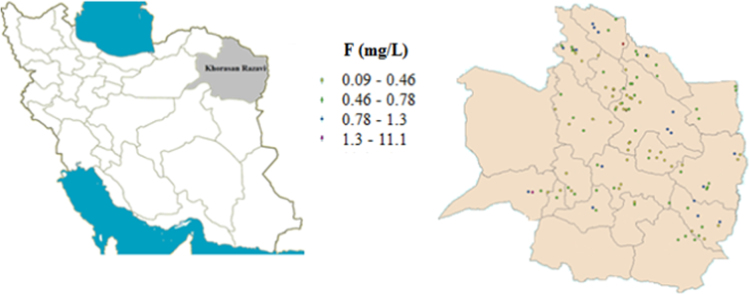
The study area.

The fluoride (F) data in this report obtained from the monitoring of the drinking water distribution networks of Khorasan Razavi province during 2016–2017. Samples taken from 112 different locations and analyzed for the F concentration by the DR-5000 spectrophotometer, according to the standard methods for the examination of water and wastewater (SPADNS Method) [1]. The study area is shown in Fig. 1. Table 2, Table 3 show the F levels in the drinking water supplies of municipal and rural areas, respectively.

**Table 1:** The constant used for the calculation of human health risk assessment parameters.

**Factor**	**Men**	**Women**	**Children**	**Unit**
**WI**	2	2	1	Litter/day
**F**	365	365	365	Day
**D**	40	40	6	A/life time
**BW**	78	65	14.5	Kg
**T**	14,600	14,600	2190	–

**Table 2:** The CDI and HQ values for F in municipal samples in Khorasan Razavi, Iran.

**Nos.**	**Fluoride (mg L**^**−1**^)	**CDI**			**HQ**		
		**Men**	**Women**	**Children**	**Men**	**Women**	**Children**
1	0.95	2.44E−02	6.25E−04	1.60E−05	4.06E−01	1.04E−02	2.67E−04
2	0.62	1.59E−02	4.08E−04	1.05E−05	2.65E−01	6.79E−03	1.74E−04
3	1.70	1.59E−02	4.08E−04	1.05E−05	2.65E−01	6.79E−03	1.74E−04
4	1.05	2.69E−02	6.90E−04	1.77E−05	4.49E−01	1.15E−02	2.95E−04
5	0.50	1.28E−02	3.29E−04	8.43E−06	2.14E−01	5.48E−03	1.40E−04
6	0.70	1.79E−02	4.60E−04	1.18E−05	2.99E−01	7.67E−03	1.97E−04
7	1.18	3.03E−02	7.76E−04	1.99E−05	5.04E−01	1.29E−02	3.32E−04
8	0.38	9.74E−03	2.50E−04	6.41E−06	1.62E−01	4.16E−03	1.07E−04
9	0.63	1.62E−02	4.14E−04	1.06E−05	2.69E−01	6.90E−03	1.77E−04
10	0.27	6.92E−03	1.78E−04	4.55E−06	1.15E−01	2.96E−03	7.59E−05
11	0.21	5.38E−03	1.38E−04	3.54E−06	8.97E−02	2.30E−03	5.90E−05
12	0.09	2.31E−03	5.92E−05	1.52E−06	3.85E−02	9.86E−04	2.53E−05
13	0.25	6.41E−03	1.64E−04	4.21E−06	1.07E−01	2.74E−03	7.02E−05
14	0.57	1.46E−02	3.75E−04	9.61E−06	2.44E−01	6.25E−03	1.60E−04
15	0.13	3.33E−03	8.55E−05	2.19E−06	5.56E−02	1.42E−03	3.65E−05
16	0.52	1.33E−02	3.42E−04	8.77E−06	2.22E−01	5.70E−03	1.46E−04
17	0.47	1.21E−02	3.09E−04	7.92E−06	2.01E−01	5.15E−03	1.32E−04
18	0.56	1.44E−02	3.68E−04	9.44E−06	2.39E−01	6.14E−03	1.57E−04
19	0.73	1.87E−02	4.80E−04	1.23E−05	3.12E−01	8.00E−03	2.05E−04
20	0.64	1.64E−02	4.21E−04	1.08E−05	2.74E−01	7.01E−03	1.80E−04
21	0.23	5.90E−03	1.51E−04	3.88E−06	9.83E−02	2.52E−03	6.46E−05
22	0.50	1.28E−02	3.29E−04	8.43E−06	2.14E−01	5.48E−03	1.40E−04
23	0.21	5.38E−03	1.38E−04	3.54E−06	8.97E−02	2.30E−03	5.90E−05
24	0.42	1.08E−02	2.76E−04	7.08E−06	1.79E−01	4.60E−03	1.18E−04
25	0.25	6.41E−03	1.64E−04	4.21E−06	1.07E−01	2.74E−03	7.02E−05
26	0.12	3.08E−03	7.89E−05	2.02E−06	5.13E−02	1.31E−03	3.37E−05
27	0.19	4.87E−03	1.25E−04	3.20E−06	8.12E−02	2.08E−03	5.34E−05
28	0.32	8.21E−03	2.10E−04	5.39E−06	1.37E−01	3.51E−03	8.99E−05
29	0.40	1.03E−02	2.63E−04	6.74E−06	1.71E−01	4.38E−03	1.12E−04
30	0.98	2.51E−02	6.44E−04	1.65E−05	4.19E−01	1.07E−02	2.75E−04
31	0.77	1.97E−02	5.06E−04	1.30E−05	3.29E−01	8.44E−03	2.16E−04
32	0.44	1.13E−02	2.89E−04	7.42E−06	1.88E−01	4.82E−03	1.24E−04
33	0.58	1.49E−02	3.81E−04	9.78E−06	2.48E−01	6.36E−03	1.63E−04
34	0.40	1.03E−02	2.63E−04	6.74E−06	1.71E−01	4.38E−03	1.12E−04
35	0.28	7.18E−03	1.84E−04	4.72E−06	1.20E−01	3.07E−03	7.87E−05
36	0.66	1.69E−02	4.34E−04	1.11E−05	2.82E−01	7.23E−03	1.85E−04
37	0.17	4.36E−03	1.12E−04	2.87E−06	7.26E−02	1.86E−03	4.78E−05
38	0.50	1.28E−02	3.29E−04	8.43E−06	2.14E−01	5.48E−03	1.40E−04
39	0.31	7.95E−03	2.04E−04	5.23E−06	1.32E−01	3.40E−03	8.71E−05
40	0.43	1.10E−02	2.83E−04	7.25E−06	1.84E−01	4.71E−03	1.21E−04

**Table 3:** The CDI and HQ values for F in rural samples in Khorasan Razavi, Iran.

**Nos.**	**Fluoride (mg L**^**−1**^)	**CDI**			**HQ**		
		**Men**	**Women**	**Children**	**Men**	**Women**	**Children**
1	0.51	1.31E−02	3.35E−04	8.60E−06	2.18E−01	5.59E−03	1.43E−04
2	0.87	2.23E−02	5.72E−04	1.47E−05	3.72E−01	9.53E−03	2.44E−04
3	0.61	1.56E−02	4.01E−04	1.03E−05	2.61E−01	6.68E−03	1.71E−04
4	0.5	1.28E−02	3.29E−04	8.43E−06	2.14E−01	5.48E−03	1.40E−04
5	0.88	2.26E−02	5.79E−04	1.48E−05	3.76E−01	9.64E−03	2.47E−04
6	0.39	1.00E−02	2.56E−04	6.57E−06	1.67E−01	4.27E−03	1.10E−04
7	0.36	9.23E−03	2.37E−04	6.07E−06	1.54E−01	3.94E−03	1.01E−04
8	0.16	4.10E−03	1.05E−04	2.70E−06	6.84E−02	1.75E−03	4.50E−05
9	0.69	1.77E−02	4.54E−04	1.16E−05	2.95E−01	7.56E−03	1.94E−04
10	0.4	1.03E−02	2.63E−04	6.74E−06	1.71E−01	4.38E−03	1.12E−04
11	0.51	1.31E−02	3.35E−04	8.60E−06	2.18E−01	5.59E−03	1.43E−04
12	0.89	2.28E−02	5.85E−04	1.50E−05	3.80E−01	9.75E−03	2.50E−04
13	0.71	1.82E−02	4.67E−04	1.20E−05	3.03E−01	7.78E−03	1.99E−04
14	0.2	5.13E−03	1.31E−04	3.37E−06	8.55E−02	2.19E−03	5.62E−05
15	0.38	9.74E−03	2.50E−04	6.41E−06	1.62E−01	4.16E−03	1.07E−04
16	0.52	1.33E−02	3.42E−04	8.77E−06	2.22E−01	5.70E−03	1.46E−04
17	1.3	3.33E−02	8.55E−04	2.19E−05	5.56E−01	1.42E−02	3.65E−04
18	0.7	1.79E−02	4.60E−04	1.18E−05	2.99E−01	7.67E−03	1.97E−04
19	0.72	1.85E−02	4.73E−04	1.21E−05	3.08E−01	7.89E−03	2.02E−04
20	0.45	1.15E−02	2.96E−04	7.59E−06	1.92E−01	4.93E−03	1.26E−04
21	0.94	2.41E−02	6.18E−04	1.58E−05	4.02E−01	1.03E−02	2.64E−04
22	0.41	1.05E−02	2.70E−04	6.91E−06	1.75E−01	4.49E−03	1.15E−04
23	0.74	1.90E−02	4.87E−04	1.25E−05	3.16E−01	8.11E−03	2.08E−04
24	0.61	1.56E−02	4.01E−04	1.03E−05	2.61E−01	6.68E−03	1.71E−04
25	0.36	9.23E−03	2.37E−04	6.07E−06	1.54E−01	3.94E−03	1.01E−04
26	0.42	1.08E−02	2.76E−04	7.08E−06	1.79E−01	4.60E−03	1.18E−04
27	46	1.18E+00	3.02E−02	7.75E−04	1.97E+01	5.04E−01	1.29E−02
28	0.63	1.62E−02	4.14E−04	1.06E−05	2.69E−01	6.90E−03	1.77E−04
29	0.83	2.13E−02	5.46E−04	1.40E−05	3.55E−01	9.09E−03	2.33E−04
30	0.89	2.28E−02	5.85E−04	1.50E−05	3.80E−01	9.75E−03	2.50E−04
31	0.92	2.36E−02	6.05E−04	1.55E−05	3.93E−01	1.01E−02	2.58E−04
32	0.72	1.85E−02	4.73E−04	1.21E−05	3.08E−01	7.89E−03	2.02E−04
33	0.61	1.56E−02	4.01E−04	1.03E−05	2.61E−01	6.68E−03	1.71E−04
34	0.44	1.13E−02	2.89E−04	7.42E−06	1.88E−01	4.82E−03	1.24E−04
35	0.58	1.49E−02	3.81E−04	9.78E−06	2.48E−01	6.36E−03	1.63E−04
36	0.6	1.54E−02	3.94E−04	1.01E−05	2.56E−01	6.57E−03	1.69E−04
37	0.5	1.28E−02	3.29E−04	8.43E−06	2.14E−01	5.48E−03	1.40E−04
38	0.29	7.44E−03	1.91E−04	4.89E−06	1.24E−01	3.18E−03	8.15E−05
39	0.49	1.26E−02	3.22E−04	8.26E−06	2.09E−01	5.37E−03	1.38E−04
40	0.37	9.49E−03	2.43E−04	6.24E−06	1.58E−01	4.05E−03	1.04E−04
41	0.23	5.90E−03	1.51E−04	3.88E−06	9.83E−02	2.52E−03	6.46E−05
42	0.62	1.59E−02	4.08E−04	1.05E−05	2.65E−01	6.79E−03	1.74E−04
43	0.72	1.85E−02	4.73E−04	1.21E−05	3.08E−01	7.89E−03	2.02E−04
44	0.46	1.18E−02	3.02E−04	7.75E−06	1.97E−01	5.04E−03	1.29E−04
45	0.66	1.69E−02	4.34E−04	1.11E−05	2.82E−01	7.23E−03	1.85E−04
46	1.1	2.82E−02	7.23E−04	1.85E−05	4.70E−01	1.21E−02	3.09E−04
47	0.38	9.74E−03	2.50E−04	6.41E−06	1.62E−01	4.16E−03	1.07E−04
48	0.78	2.00E−02	5.13E−04	1.31E−05	3.33E−01	8.55E−03	2.19E−04
49	0.77	1.97E−02	5.06E−04	1.30E−05	3.29E−01	8.44E−03	2.16E−04
50	0.16	4.10E−03	1.05E−04	2.70E−06	6.84E−02	1.75E−03	4.50E−05
51	0.56	1.44E−02	3.68E−04	9.44E−06	2.39E−01	6.14E−03	1.57E−04
52	0.48	1.23E−02	3.16E−04	8.09E−06	2.05E−01	5.26E−03	1.35E−04
53	0.2	5.13E−03	1.31E−04	3.37E−06	8.55E−02	2.19E−03	5.62E−05
54	0.87	2.23E−02	5.72E−04	1.47E−05	3.72E−01	9.53E−03	2.44E−04
55	0.69	1.77E−02	4.54E−04	1.16E−05	2.95E−01	7.56E−03	1.94E−04
56	0.76	1.95E−02	5.00E−04	1.28E−05	3.25E−01	8.33E−03	2.14E−04
57	0.55	1.41E−02	3.62E−04	9.27E−06	2.35E−01	6.03E−03	1.55E−04
58	0.26	6.67E−03	1.71E−04	4.38E−06	1.11E−01	2.85E−03	7.31E−05
59	0.72	1.85E−02	4.73E−04	1.21E−05	3.08E−01	7.89E−03	2.02E−04
60	0.44	1.13E−02	2.89E−04	7.42E−06	1.88E−01	4.82E−03	1.24E−04
61	0.34	8.72E−03	2.24E−04	5.73E−06	1.45E−01	3.73E−03	9.55E−05
62	0.38	9.74E−03	2.50E−04	6.41E−06	1.62E−01	4.16E−03	1.07E−04
63	0.39	1.00E−02	2.56E−04	6.57E−06	1.67E−01	4.27E−03	1.10E−04
64	0.58	1.49E−02	3.81E−04	9.78E−06	2.48E−01	6.36E−03	1.63E−04
65	0.97	2.49E−02	6.38E−04	1.64E−05	4.15E−01	1.06E−02	2.73E−04
66	1.1	2.82E−02	7.23E−04	1.85E−05	4.70E−01	1.21E−02	3.09E−04
67	0.93	2.38E−02	6.11E−04	1.57E−05	3.97E−01	1.02E−02	2.61E−04
68	0.6	1.54E−02	3.94E−04	1.01E−05	2.56E−01	6.57E−03	1.69E−04
69	0.57	1.46E−02	3.75E−04	9.61E−06	2.44E−01	6.25E−03	1.60E−04
70	0.22	5.64E−03	1.45E−04	3.71E−06	9.40E−02	2.41E−03	6.18E−05
71	0.71	1.82E−02	4.67E−04	1.20E−05	3.03E−01	7.78E−03	1.99E−04
72	0.78	2.00E−02	5.13E−04	1.31E−05	3.33E−01	8.55E−03	2.19E−04

## 2 Experimental design, materials, and methods

Fluoride (F) is a critical ion in drinking water. The unusual nature of F in posing detrimental health effects at excess or deficient intake, motivated researchers to conduct numerous studies on both F level in drinking waters and excessive F removal from contaminated streams [2], [3], [4], [5].

Human health risk assessment for water contaminants estimate the nature and probability of adverse health effects for the population who receive the chemicals from drinking water. It provides a systematic approach for developing management strategies to supply safe drinking water. The data obtained from the analysis of samples for F in 112 locations across the Khorasan Razavi province undergo a health risk assessment for non-cancer effects. The following equation (Eq. 1) was used to calculate the non-carcinogenic health risk [6], [7]:

(1) $$\mathrm{HQ}=\frac{\mathrm{CDI}}{\mathrm{RfD}}$$

where HQ is non-carcinogenic risk quotient. CDI and RfD are chronic daily intake (mg kg^−^^1^day^−^^1^) and reference dose (mg kg^−1^day^−1^), respectively. The intake reference dose for F is 0.06 mg kg^−1^day^−1^ [8]. The following Eq. (2) is used to calculate the CDI:

(2) $$\mathrm{CDI}=\frac{C_{W}\times W_{I}\times F\times D}{W\times T}$$

where *C_W_*, *W_I_*, *F*, *D*, *W*, and *T* are F content in drinking water (as measured), water intake, exposure frequency, exposure duration, body weight, and average life time, respectively [4]. The constants used for the above formula are shown in Table 1. An HQ value more than one will show a substantial risk, where the higher the value, the greater the likelihood of adverse non-carcinogenic health effects. The RfD value for F^−^ was 0.6 mg kg^−1^day^−1^. The mean F concentration for municipal and rural samples were 0.74 and 0.59 mg L^−1^, respectively. The concentration of F in municipal and rural samples was 1.7 to 0.09 mg L^−1^ and 1.3 to 0.16 mg L^−1^, respectively. Based on the analyzed data, the mean value of the CDI for F in municipal samples for men, women, and children was 1.3×10^−2^ (4.36×10^−2^ to 2.31×10^−2^), 3.34×10^−4^ (1.12×10^−3^ to 5.92×10^−5^), and 8.56×10^−6^ (2.87×10^−5^ to 1.52×10^−6^) mg kg^−1^day^−1^, respectively (Table 2). Furthermore, the mean value of the CDI for F in rural samples for men, women, and children was 1.51×10^−2^ (3.33×10^−2^ to 4.1×10^−3^), 3.88×10^−4^ (8.55×10^−4^ to 1.05×10^−4^), and 9.96×10^−6^ (2.19×10^−5^ to 2.76×10^−6^) mg kg^−1^day^−1^, respectively (Table 3). The mean HQ value of F for men, women, and children in municipal samples was 3.17×10^−1^ (4.74 to 3.85×10^−2^), 8.14×10^−3^ (1.22×10^−1^ to 9.86×10^−4^), and 2.09×10^−5^ (3.12×10^−3^ to 2.53×10^−5^), respectively. HQ of more than one, indicated that the F level is unacceptably high and non-cancer negative health consequences is highly probable. This must be considered for health care decision makers in water supply industry.
