# Supplementary material for: Data on fluoride concentration and health risk assessment of drinking water in Khorasan Razavi province, Iran
Source: Data Brief. 2018 Apr 21;18:1596–601. doi: 10.1016/j.dib.2018.04.045 (PMC5997963; doi:10.1016/j.dib.2018.04.045)
Supplement: Supplementary file 1 — Supplementary material [file mmc1.docx]

**Data Article**

**Data on fluoride concentration and health risk assessment of drinking water in Khorasan Razavi province, Iran**

Mansour Ghaderpoori^1, 2^, Ali Asghar Najafpoor^3, 4^, Afshin Ghaderpoury^5^, Mahmoud Shams ^* 3, 4^

1. Nutritional Health Research Center, Lorestan University of Medical Sciences, Khorramabad, Iran
2. Department of environmental health engineering, School of Health and Nutrition, Lorestan University of medical sciences, Khorramabad, Iran (ghaderpoori.m@lums.ac.ir)
3. Department of Environmental Health Engineering, School of Health, Mashhad University of Medical Sciences, Mashhad, Iran
4. Health Sciences Research Center, Department of Environmental Health, Mashhad University of Medical Sciences, Mashhad, Iran
5. Students Research Committee, Shahid Beheshti University of Medical Sciences, Tehran, Iran ([ghaderpoury_a@yahoo.com](mailto:ghaderpoury_a@yahoo.com))

**Corresponding author Contact email**: Mahmoud Shams ([Shamsmh@mums.ac.ir](mailto:Shamsmh@mums.ac.ir))

**Conflict of Interest:**

The authors of this article declare that they have no conflict of interests.
